# Mechanistic Insights into the Formation of Monodisperse Porous Poly(GMA-*co*-EDMA) Microspheres

**DOI:** 10.3390/polym18151813

**Published:** 2026-07-24

**Authors:** Alexandra Wagner, Julia Schwabe, Frank Wackenhut, Julia C. Steinbach, Ashutosh Mukherjee, Andreas Kandelbauer, Hermann A. Mayer, Marc Brecht

**Affiliations:** 1 Process Analysis and Technology (PA&T), Reutlingen Research Institute, Reutlingen University, Alteburgstrasse 150, 72762 Reutlingen, Germany; alexandra.wagner@pct-chemie.de (A.W.); julia.schwabe@reutlingen-university.de (J.S.); julia.steinbach@de.bosch.com (J.C.S.); ashutosh.mukherjee@reutlingen-university.de (A.M.); andreas.kandelbauer@reutlingen-university.de (A.K.); 2 Institute of Inorganic Chemistry, University of Tübingen, Auf der Morgenstelle 18, 72076 Tübingen, Germany; hermann.mayer@uni-tuebingen.de

**Keywords:** p(GMA-*co*-EDMA), porous polymer microspheres, 2D confocal Raman spectroscopy, SEM, particle formation mechanism

## Abstract

Monodisperse porous polymer spheres are functional materials with attractive properties such as high cohesive strength, strong adsorptivity, and a high degree of surface functionalization due to their large specific surface area. They are widely used in various fields, including biomedicine, instrumental analytics as stationary phases in HPLC columns, and sensor technology. In this work, the formation mechanism of porous poly(glycidyl methacrylate-co-ethylene dimethacrylate) (p(GMA-*co*-EDMA)) particles was investigated in a time-resolved experiment using scanning electron microscopy (SEM) and two-dimensional confocal Raman spectroscopy. Data analysis revealed that the particles are formed via rapidly reacting anisotropic Janus-like intermediates that assemble into large agglomerates of different morphologies. Spectral unmixing of the Raman data enabled the determination of relative concentration changes in the reactants over time. This study uncovers a pool of previously unknown anisotropic particle species that offer new opportunities for subsequent functionalization and material design.

## 1. Introduction

Spherical monodisperse micron-sized polymeric particles have been utilized in numerous applications for decades. They are of great interest across multiple scientific fields and represent highly attractive materials in biomedical research, where they are used as drug delivery and biosensing systems [1,2,3,4,5,6]. Besides the scientific interest, there are many applications, e.g., in separation processes like chromatography [7,8,9,10], in catalysis [11,12] or as ion exchange materials [13,14].

Polymer particles are generally synthesized via heterogeneous polymerization processes that exploit the immiscibility of two or more liquid phases. One of these approaches is seed-swelling polymerization, a two-step procedure in which polymer seed particles absorb monomers and porogens—up to 100 times their own volume—forming emulsion droplets. In a subsequent reaction, the adsorbed molecules polymerize inside the swollen particle [15]. Spherical particle formation is favored due to interfacial tension effects acting at the surface of the emulsion droplets. The final particle size, typically ranging from 1 to 200 µm, depends strongly on the preparation conditions of the seed materials, and synthesis parameters can be adjusted to tailor the resulting material properties.

Besides general process variables such as temperature, pressure, and stirring speed, the chemical composition of the system significantly influences particle characteristics. Of particular interest are particles bearing functional groups, such as carboxylic acids, esters, aldehydes, ketones, or epoxides, which enable subsequent covalent attachment of biomolecules including proteins, nucleic acids, enzymes, antibodies, or fluorescent dyes [16,17]. In this study, glycidyl methacrylate (GMA) and ethylene dimethacrylate (EDMA) were used as monomers to synthesize poly(glycidyl methacrylate-co-ethylene dimethacrylate) (p(GMA-*co*-EDMA)) particles. Due to the terminal epoxy group of GMA, the resulting particles can be further functionalized under mild reaction conditions [18,19,20,21].

In addition to the monomers forming the polymer backbone, seed particles and porogens can also be varied to modify particle properties. Here, polystyrene (PS) beads were used as seed particles, while porogens were selected to induce porosity. Their effect is governed primarily by miscibility relationships between seeds, monomers, and porogens. Solubility parameters provide a qualitative predictive criterion for particle morphology, porosity, and pore volume, enabling controlled synthesis of micro-, meso- or macroporous structures. The three-dimensional Hansen solubility parameter model offers the most reliable description of solubility behavior [22]. According to this model, each substance is characterized by three Hansen parameters which correspond to the three dominant existing forces: London dispersion, dipole–dipole, and hydrogen bonding. They define a point in the three-dimensional Hansen space (R_a_) [23]. These interactions correlate with the dispersive solubility parameter (δ_D_), the polar solubility parameter (δ_P_), and the H-bonding solubility parameter (δ_H_), respectively. The proximity of two coordinates correlates with their mutual solubility [7].

Solvent suitability can furthermore be estimated using the relative energy difference (RED), defined as the ratio of the R_a_ distance to the Hansen interaction radius R_0_. Good solvents exhibit RED values below 1. Solvents around 1 are found to only partly dissolve the solute (or swell it), while non-solvents show values above 1 [23]. Good solvents for PS seed-based synthesis of spherical, porous, and monodisperse particles include dibutyl phthalate (RED = 0.59), toluene (RED = 0.65), and cyclohexanol (RED = 0.96) [24]. In contrast, poor solvents such as 1-hexanol (RED = 1.07) cause solubility changes during polymerization, leading to phase separation and formation of biphasic particles [25,26].

In this work, porogen-induced immiscibility effects and the resulting biphasic particle formation were exploited to establish a synthesis route in which Janus particles (JPs) occur as intermediates, ultimately yielding homogeneous particles with a narrow size distribution and high porosity. Here, 1-hexanol was identified as a suitable porogen to induce controlled phase separation [25]. Because 1-hexanol exhibits low solubility within the PS seeds, an early phase separation occurs, and its diffusion rate decreases during polymerization due to continuously changing component concentrations. This dynamic leads to the formation of biphasic particles, enabling the straightforward synthesis of regular non-spherical JPs, which are otherwise difficult to obtain.

Furthermore, we were able to identify several intermediate structures with different properties, including shape, size and surface morphology, that occur during the polymerization and thus explain the formation mechanism of p(GMA-*co*-EDMA) particles. The chemical composition and molecular distribution of all intermediates were characterized using confocal Raman imaging [25]. The complementary combination of Raman imaging and scanning electron microscopy (SEM) provides detailed insight into both molecular composition and physical surface characteristics, enabling step-by-step monitoring of particle formation and offering a deeper mechanistic understanding of the underlying process [25].

## 2. Materials and Methods

### 2.1. Reagents and Chemicals

Styrene (99%) was purchased from Fisher Scientific GmbH (Schwerte, Germany). Polyvinylpyrrolidone K30 (PVP) and benzoyl peroxide (BPO, 75%) and Ethylene dimethacrylate (EDMA) were obtained from Sigma-Aldrich Chemie GmbH (Taufkirchen, Germany). Ethanol (96%) was supplied from VWR Chemicals (Darmstadt, Germany). Glycidyl methacrylate (GMA), and sodium dodecyl sulfate (SDS) were purchased from abcr GmbH (Karlsruhe, Germany). 1-hexanol and sodium nitrite (NaNO_2_) were supplied from Merck GmbH (Darmstadt, Germany).

### 2.2. Preparation of Monodisperse p(GMA-co-EDMA) Microspheres

Monodisperse porous p(GMA-*co*-EDMA) microspheres were prepared by a two-step seed swelling polymerization process. In a first step, 1 g of polystyrene (PS) seed particles were dispersed in 30 mL of deionized water by sonication for 10 min and subsequently stirred at 200 rpm for 30 min at 30 °C.

Separately, an emulsion containing glycidyl methacrylate (GMA, 7 mL), ethylene glycol dimethacrylate (EDMA, 7 mL), sodium dodecyl sulfate (SDS, 0.3 g), poly(vinylpyrrolidone) (PVP, 3 g), 1-hexanol (14 mL) as porogen, benzoyl peroxide (BPO, 0.56 g) as initiator, and 250 mL of deionized water was prepared by homogenization at 10,000 rpm for 10 min. This emulsion was added to the PS seed suspension and stirred continuously at 200 rpm for 24 h in a preheated oil bath at 30 °C to allow adsorption and swelling of the seeds with the monomers, porogen, and initiator.

After the swelling period, polymerization was initiated by increasing the temperature to 70 °C and maintaining it for 24 h. The reaction temperature was monitored in the oil bath. Since polymerization was initiated after increasing the temperature to 70 °C, minor variations of approximately 1–2 °C during the initial swelling stage are not expected to significantly affect the particle morphology. However, temperature remains an important parameter influencing polymerization kinetics and phase separation behavior.

To investigate the reaction progress, samples were withdrawn at short time intervals. Each sample vial contained 1 mL of sodium nitrite (NaNO_2_, c = 0.2 mg/mL) to inhibit further polymerization. The collected samples were alternately washed three times with deionized water and ethanol and subsequently stored in ethanol for further analysis.

### 2.3. Sample Collection

To investigate the reaction mechanism and reveal individual intermediates, a total of 50 samples were collected at different time intervals. Five samples were taken after the swelling period while heating from 30 to 70 °C. After reaching 70 °C, a sample was taken every minute for 45 min. For this purpose, 2 mL of the reaction mixture was removed and placed in a snap lid jar with 1 mL NaNO_2_ (c = 0.2 mg/mL) added to quench the reaction and “freeze” the current polymerization progress [27]. The intermediates were washed with deionized water and ethanol (99%) for three times and left in ethanol until further investigations.

### 2.4. Imaging and Spectroscopy

Light microscopic images, Raman spectra and Raman maps were acquired with a confocal Raman microscope (Oxford Instruments WiTec alpha300 RA&S, Ulm, Germany) equipped with an air objective (Carl Zeiss, Oberkochen, Germany; EC Epiplan-Neofluar DIC M27, 100×, NA = 0.90) providing a lateral resolution of 360 nm. The system includes a UHTS 300 spectrometer, Oxford Instruments Andor, connected via a 100 µm (NA = 0.12) multimode fiber and a thermoelectrically cooled CCD and EMCCD camera (DU970N-BV). All Raman experiments were conducted using a 532 nm laser with a nominal output power of approx. 39 mW and a grating with 600 lines/mm. Data acquisition and processing were performed with WITec Control 5.3 software [28].

For Raman measurements, a droplet (5 µL) of the mixed dispersion sample was deposited onto a cleaned sapphire microscope slide and allowed to evaporate. All experiments were performed under ambient conditions. Sapphire was selected as the substrate due to its very low background signal in the spectral region of interest and the absence of spectral overlap with the sample, making it ideal for Raman analysis of these particles.

### 2.5. Scanning Electron Microscopy

Polymerization intermediates were characterized using scanning electron microscopy (SEM, PHENOM^®^, EM-10001, PHENOM-World B.V., Eindhoven, The Netherlands). Samples were placed on a silica wafer, which was mounted onto a SEM sample stub (diameter: 12.7 mm) using silver adhesive. All samples were sputtered with a gold layer of approx. 70 nm thickness before analysis. Imaging was performed at various magnifications.

### 2.6. Particle Size Distribution

Particle size distributions were determined from SEM images of the respective intermediates using ImageJ (version 1.52a) for image analysis. Statistical parameters—including total number of analyzed particles, mean, median, standard deviation, and minimum/maximum particle diameter—were calculated with OriginPro^®^2021b and are summarized in Table S1 (Supplementary Information).

### 2.7. Spectral Unmixing

Spectral unmixing was applied to gain deeper insight into the Raman spectra acquired from 2D Raman maps. This method calculates the contribution of individual pure components to each Raman spectrum in the dataset. The spectral region from 1550 cm^−1^ to 1750 cm^−1^, containing an intense C=C band at 1600 cm^−1^ correlating with PS and a characteristic p(GMA-*co*-EDMA) band at 1720 cm^−1^, was selected to differentiate between PS and p(GMA-*co*-EDMA). The Raman spectra of the pure components were normalized to values between 0 and 1, and the full 2D dataset was likewise normalized. Calculations were performed by solving D = C Sᵀ, where D is the data matrix, C the coefficient matrix, and Sᵀ the transposed pure component spectra. This provides abundance maps indicating the relative contribution of each pure component to each pixel spectrum.

## 3. Results

**Figure 1:**
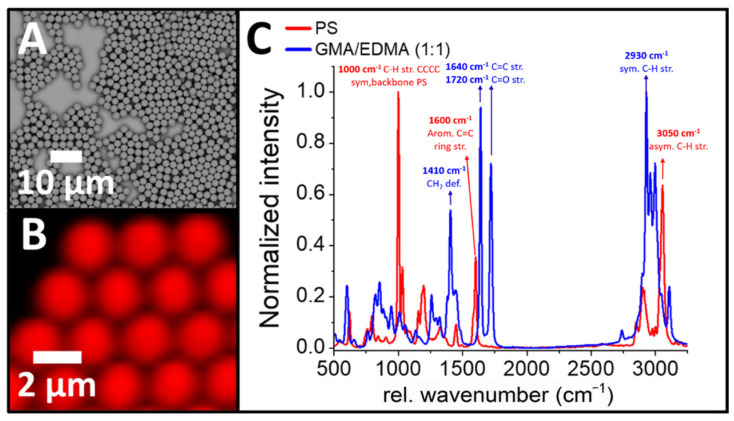
(**A**) PS particles imaged by SEM with a diameter of 2.13 ± 0.06 µm and (**B**) the corresponding Raman image. (**C**) Averaged (10 spectra) and normalized Raman spectra of PS particles (red) and GMA:EDMA (1:1) (blue) in the range 500–3300 cm^−1^.

The initial point is the use of spherical and highly monodisperse PS seed particles, as shown by SEM and confocal microscopy images in Figure 1A and 1B, respectively.

The mean diameter of the PS seeds is 2.13 ± 0.06 µm, which is identical to the median diameter of 2.13 µm (Supporting Information, Table S1). Raman spectra of the PS particles and of a GMA:EDMA mixture are shown in Figure 1C, with the most prominent vibrational Raman bands indicated. Raman spectroscopy clearly distinguishes PS and the GMA:EDMA (1:1) mixture due to their distinct band positions, particularly in the range of 1550–1750 cm^−1^. The corresponding Raman spectra of pure PS and the GMA:EDMA mixture are provided in the Supporting Information (Figure S1) [25].

**Figure 2:**
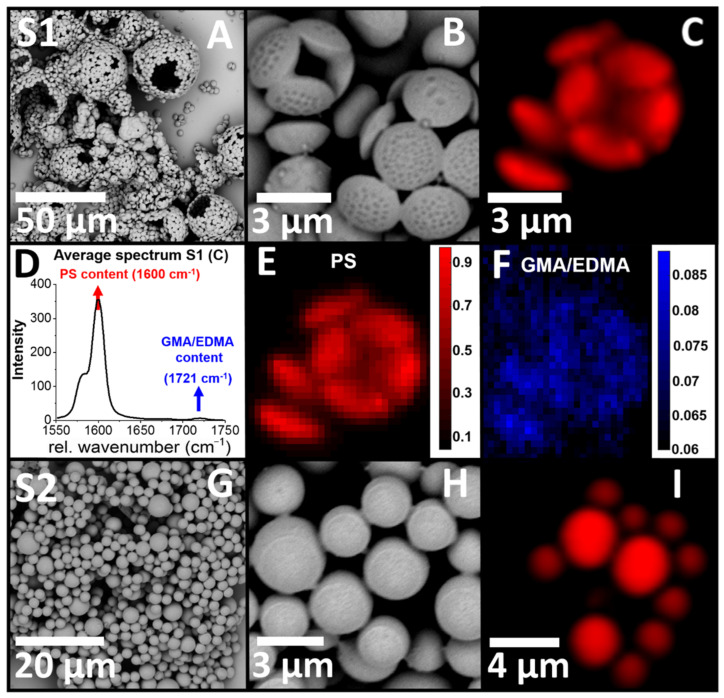
(**A**,**B**) SEM images of S1 (t = 0 min, T = 30 °C) acquired at different lateral positions and magnifications. (**C**) Confocal Raman image of S1 particles. (**D**) Mean Raman spectrum of all particles shown in (**C**) in the range 1550 cm^−1^–1750 cm^−1^. (**E**,**F**) Abundance maps of PS and GMA/EDMA, showing the contribution of the respective component in each pixel. (**G**) Overview image of S2 (t = 35 min, T = 70 °C), showing particles that have changed their shape and become more spherical. (**H**) High-magnification SEM image; a small cap is clearly visible on the particle. (**I**) Corresponding Raman image, indicating that the particles mainly consist of PS.

Following a 24 h swelling period, the particles undergo a shape transformation from spherical (Figure 1A) to lens-shaped, as evident by SEM images of sample S1 (30 °C, t = 0 min) shown in Figure 2A,B. Notably, the lens-shaped particles are organized into large, spherical agglomerates (Figure 2A), that are consistently observed throughout the entire sample and exhibit a broad size distribution. Each agglomerate consists of a monolayer of individual lens-shaped particles, most likely formed at the surface of a GMA/EDMA monomer droplet (Figure 2A). This model explains the observed shape transformation: a reduction in interfacial tension at the three-phase boundary between the PS particle, water, and GMA/EDMA. This leads to a finite contact angle, resulting in a stable, energetically favored lens-shaped geometry [29,30,31,32,33]. One side of each lens-shaped particle is smooth, whereas the opposite side exhibits a textured surface with dents of varying sizes (Figure 2B). The major axis diameter of the elliptical particles has a mean value of 3.02 ± 0.12 µm, identical to the median diameter of 3.02 µm (Supporting Information, Table S1). The confocal Raman image of S1 (Figure 2C) shows a small agglomerate of lens-shaped particles. The corresponding average Raman spectrum in the spectral range between 1550 cm^−1^ and 1750 cm^−1^ (Figure 2D) is dominated by PS, with only a weak signal at 1721 cm^−1^ indicating the presence of GMA/EDMA. Using spectral unmixing, the contributions of PS and GMA/EDMA can be clearly distinguished, as shown in the corresponding abundance maps in Figure 2E,F. Upon heating the reaction mixture to 70 °C, no changes in particle shape or size are observed after 30 min (Supporting Information, Figure S2). S2 was collected after 35 min at 70 °C. SEM images (Figure 2G,H) reveal a transition of the particles from a lens-like to a spherical shape, accompanied by a decrease in the mean diameter to 2.76 ± 1.31 µm, resulting in a loss of monodispersity. In addition, the particle morphology indicates the presence of two phases (Figure 2H). The confocal Raman image of S2 (Figure 2I) shows no detectable GMA/EDMA signal, which would appear in blue, and only a dominant PS signal is observed. The confocal Raman images and the corresponding raw Raman spectra of samples S1 and S2 shown in the Supporting Information (Figure S1) confirm that no spectrally distinguishable p(GMA-*co*-EDMA) is present in the particles at this stage.

**Figure 3:**
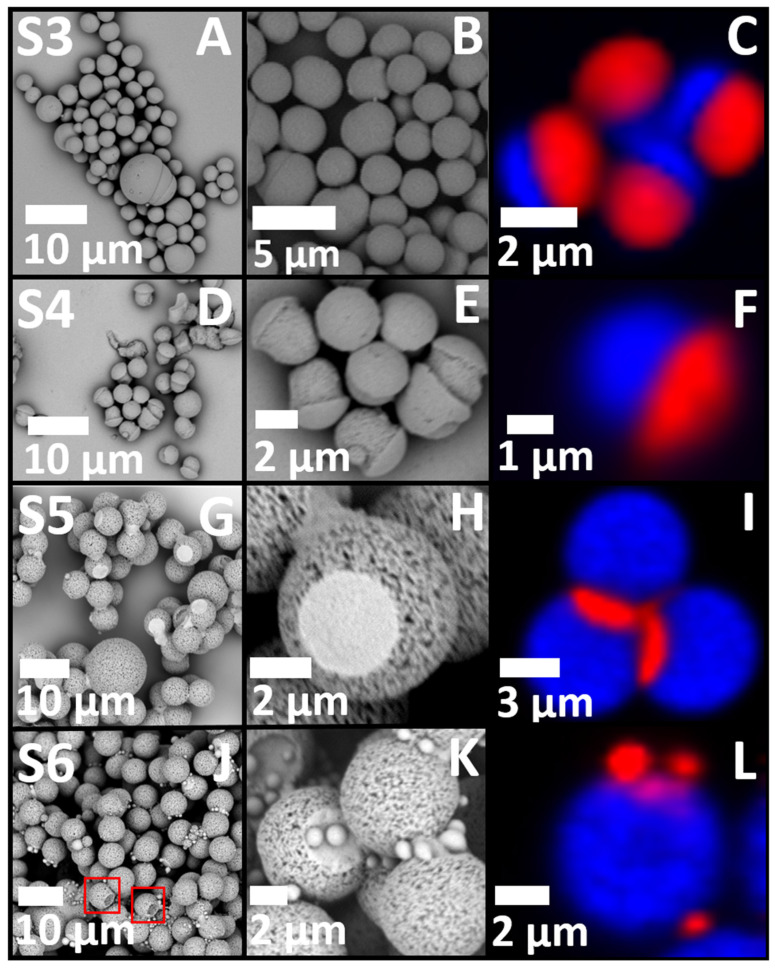
SEM overview and high-magnification images of S3–S6, together with the corresponding Raman images. (**A**) SEM overview image of S3 acquired 40 min after the start of the reaction. (**B**) Representative high-magnification SEM image of S3 showing particle morphology in greater detail. (**C**) Corresponding Raman image of S3 with PS shown in red and GMA/EDMA in blue. (**D**,**E**) SEM overview and high-magnification images of S4, acquired 45 min after reaction start. (**F**) Corresponding Raman image of S4. (**G**–**I**) SEM overview image, enlarged SEM image, and corresponding Raman image of S5; the sample was taken after 1 h. (**J**–**L**) SEM overview and high-magnification images and the corresponding Raman image of S6; the sample was taken after 24 h. The red box in (**J**) highlights particles without a PS cap, showing a hole-like opening instead.

S3 was collected after a total reaction time of 40 min. SEM images (Figure 3A,B) show that the particles are polydisperse, with an average diameter of 2.84 ± 0.52 µm.

Remarkably, after only 5 more min at 70 °C, the particle morphology has changed substantially. Compared to S2, the particles in sample S3 develop an oval shape with distinct phase boundaries. The surface morphology of both particle regions appears to be similarly smooth (Figure 3A,B). One side of the particle is slightly more bulbous than the other, giving the impression that the smaller particle section is growing out of the larger one. The corresponding Raman image (Figure 3C) reveals that these particles consist of two chemically different components that can be spectrally separated via True Component Analysis (WiTec Project 5.3) [34,35,36,37]. The larger, more bulbous region mainly consists of PS, whereas the smaller outgrowing region is composed of p(GMA-*co*-EDMA). In contrast to the monomers GMA and EDMA, the polymerized network p(GMA-*co*-EDMA) is no longer soluble in the PS seed particles. Due to this solubility shift, phase separation occurs within the particle, resulting in the formation of distinct PS and a p(GMA-*co*-EDMA) domains with sharp phase boundaries [29]. Samples S3–S6 represent JPs of different sizes and geometries. As the reaction progresses, the phase separation becomes more pronounced until phase inversion occurs. This phase inversion is observed for the first time in the SEM images of S4, acquired after 45 min at 70 °C (Figure 3D,E). The SEM images show that the two particle domains differ in morphology. The more elongated, narrow region is porous, whereas the broader cap is not. The Raman image confirms that the p(GMA-*co*-EDMA) phase has increased substantially in volume relative to the PS phase (Figure 3F). Their size and shape correspond to the individual particles; the fragments consist of a monolayer of particles connected via fused PS caps. In S5, collected 60 min after the start of the reaction, spherical particles have already formed. These consist of a porous main particle with a small, flat, non-porous PS cap (Figure 3G,H). Notably, PS appears viscous when located between two particles, particularly in the pair connected by the PS cap marked by the red rectangle in Figure 3G. The Raman image indicates that the cap is not merely a thin surface layer but also extends into the particle interior (Figure 3I). The particles are monodisperse with an average diameter of 5.88 ± 0.19 µm. SEM images of S6, collected after terminating the reaction at 24 h, reveal spherical, monodisperse particles with an average diameter of 6.38 ± 0.19 µm (Figure 3J,K). They are approximately 0.5 µm larger compared to particles in S5 (Supporting Information, Table S1) and consist of a porous p(GMA-*co*-EDMA) main body with a flat PS cap. The cap protrudes concavely into the particle, indicating partial dissolution of PS during the 24 h period (Figure 3K). In the Raman image (Figure 3L), the cap appears pink- a mixture of blue and red- indicating that Raman signals of both PS and p(GMA-*co*-EDMA) are detected in this region, consistent with a thin interfacial layer. This interpretation is supported by the concave cap morphology. The corresponding spectrum of the overlapping area is shown in the Supporting Information (Figure S1). Compared to S5, additional small, spherical, non-porous PS particles are present in S6. These particles primarily accumulate on the PS caps and appear to be released during the reaction (Figure 3K). Moreover, particles lacking the PS cap—showing a hole-like opening instead—are repeatedly observed in S6 (Figure 3J, red rectangle).

**Figure 4:**
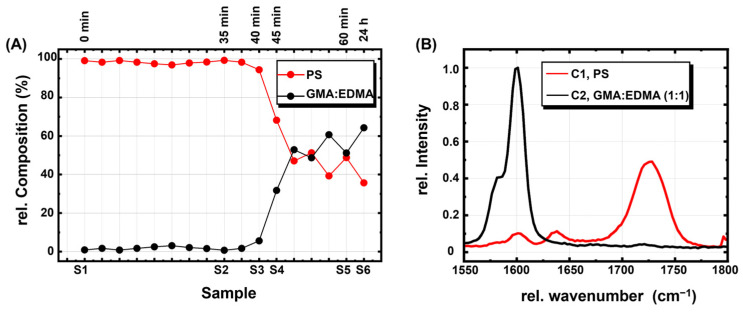
(**A**) Relative component concentrations obtained by spectral unmixing of PS (red) and GMA:EDMA (1:1, black) during polymerization. The lower x-axis indicates the analyzed samples (S1–S6), while the upper x-axis shows the corresponding reaction times. (**B**) Reference spectra of PS (C1) and GMA:EDMA (C2) in the spectral range 1550–1800 cm^−1^ with assignments of characteristic vibrational bands.

To monitor the progression of polymerization, phase separation, the increase in monomer-derived polymer, and the corresponding decrease in polystyrene over time, spectral unmixing was performed. Figure 4 shows the spectral unmixing coefficients for the polymerization progress (Figure 4A) along with the corresponding pure component spectra of PS (red, C1) and a 1:1 GMA:EDMA copolymer (black, C2), obtained after polymerization, in the spectral range of 1550–1800 cm^−1^ (Figure 4B). The characteristic band positions related to Figure 4A are summarized in Table 1. The spectra for unmixing were extracted from hyperspectral Raman images (>600 spectra), averaged, baseline-corrected, and area-normalized. For calculating the relative component concentrations, the mixed spectra of S1–S6 were decomposed into the two basic components PS (red, C1) and GMA:EDMA (black, C2) (Figure 4B). In sample S1, C1 accounts for 99.1%, confirming that the system initially consists almost entirely of PS. Up to 40 min (S3), the spectra remain dominated by PS. Thereafter, the gradual increase in C2 indicates the onset of p(GMA-*co*-EDMA) radical polymerization. After 45 min (S4), the relative GMA:EDMA-derived component reaches 31.8%, while PS still predominates (68.2%). This time window between 40 and 45 min marks the beginning of polymer network formation. Within the following 15 min, the C2 fraction further increases from 31.8% (S4) to 51.2% (S5). After 24 h (S6), the C2 concentration reaches 64.3%, indicating completion of polymerization. Approximately 35.7% PS remains embedded in the p(GMA-*co*-EDMA) matrix and in the non-porous cap. The cumulative model fit of the unmixing analysis amounts to 98.2%.

## 4. Discussion

In this work, the formation of porous poly(glycidylmethacrylate-*co*-ethylenedimethacrylate) particles based on non-porous PS was investigated. SEM was used to visualize the particle morphology, while confocal Raman spectroscopy and imaging were applied to determine the chemical position. The particles were synthesized via a two-step seed swelling polymerization process in which PS seeds were first swollen with GMA, EDMA, 1-hexanol, BPO, SDS and PVP, followed by thermal initiation of polymerization. To capture all relevant intermediate states, samples were taken at short time intervals (1 min), and polymerization in each vial was quenched with NaNO_2_. Progression of polymerization over time was analyzed by spectral unmixing (Figure 4A).

**Figure 5:**
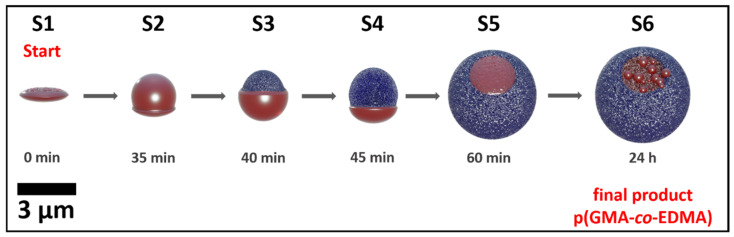
Schematic representation of the intermediates S1–S6 formed during the reaction process. Particles size correspond to those of the original SEM images. PS shown in red and GMA/EDMA in blue.

Over the 48 h reaction period, six intermediates (S1–S6) were identified and characterized. An overview of the different stages is shown in Figure 5.

The PS seeds were spherical, monodisperse (2.13 ± 0.06 µm), and exhibited characteristic Raman bands (Figure 1A,C). The intense band at 1000 cm^−1^ in the PS spectrum corresponds to the benzene ring deformation mode, indicating the presence of an aromatic system. In contrast, the Raman spectrum of a 1:1 GMA:EDMA mixture shows a C=C stretching vibration at 1640 cm^−1^ and a characteristic carbonyl vibration at 1720 cm^−1^. These distinct spectral features enable clear discrimination between PS and the monomer mixture. The first sample (S1) was taken immediately after mixing swollen PS seed particles and the previously homogenized emulsion at 30 °C. The sample consisted of lens-shaped particles that are predominantly arranged into spherical agglomerates of varying sizes (Figure 2A). Although individual particles and irregular assemblies were present (Figure 2A,B), confocal Raman imaging revealed that the structures were composed primarily of PS, despite their altered shape relative to the original seeds. A quantitative comparison of particle size distributions for PS, S1, and S6 is provided in the Supporting Information (Figure S3), showing that while the PS seeds remain highly monodisperse, the transition to lens-shaped particles in S1 is accompanied by a broader size distribution, which further increases in the final particles (S6). Spectral unmixing analysis showed that the Raman image decomposes into two pure components corresponding to PS and GMA/EDMA, demonstrating that trace amounts of monomers had already diffused into the PS matrix. The hollow appearance of agglomerates in SEM and Raman images results from sample preparation and does not reflect the true internal structure. During homogenization of GMA, EDMA, 1-hexanol, BPO, SDS, and PVP in water, a network of water-insoluble organic droplets of various sizes forms, resulting in an oil-in-water (O/W) emulsion. Because PS is insoluble in both the aqueous phase and the organic phase—particularly due to the poor solubility of 1-hexanol in PS (RED = 1.07)—the seeds preferentially migrate to the interface between the immiscible phases. Increasing temperature raises the kinetic energy and amplifies interfacial tension, promoting accumulation of PS seeds at the O/W interface as thermodynamically favored lens-shaped structures (Figure 2A,B) [30,31,32,33]. The presence of large spherical agglomerates further supports the existence of polydisperse oil droplets within the emulsion. In contrast to previously described seed swelling polymerization mechanisms, where swollen seed particles are commonly considered as individually dispersed entities that subsequently undergo polymerization and pore formation, our results reveal an earlier stage of particle–particle interaction and interfacial organization. The formation of lens-shaped PS assemblies at the oil–water interface and their subsequent transformation into Janus-like intermediates demonstrate that phase separation and particle rearrangement occur before the final polymer network is established. This observation provides new insight into the dynamic evolution of anisotropic intermediates during porous particle formation. The interfacial localization of PS at the oil–water boundary facilitates the diffusion of GMA, EDMA, BPO, and, to a limited extent, 1-hexanol into the PS particles. Partial penetration of 1-hexanol arises from its solubility in the monomers, which act as good solvents for PS according to Hansen solubility theory. Thus, 1-hexanol plays a dual role as porogen and mediator of solvent–polymer interactions, shaping both the spatial arrangement at the droplet interface and the subsequent polymerization process. This initial interfacial arrangement represents a critical step in the particle formation mechanism, as the spatial localization of PS at the oil–water interface determines the subsequent phase separation behavior during polymerization. As monomer conversion progresses, the increasing incompatibility between the growing p(GMA-*co*-EDMA) network and the PS phase drives the formation of anisotropic intermediate structures. After 35 min of polymerization (S2), two distinct structures with different surface topographies can already be observed. In addition to the smooth PS structure, a second porous structure appears, accompanied by a clear phase separation between both regions. The confocal Raman image (Figure 2I) again shows a dominance of the PS signal. SEM images reveal that the particles change their morphology from lens-shaped to spherical, which can be attributed to the diffusion of monomers into the particle interior where polymerization takes place, leading to the formation of progressively longer polymer chains. As a result, the volume of the seed particles increases and their surface tension rises.

**Figure 6:**
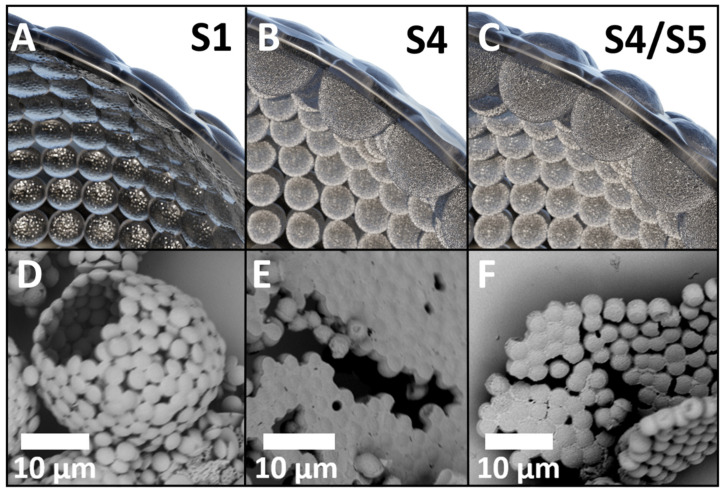
(**A**–**C**) Particle growth in a monolayer at different reaction times, visualized using Render. (**D**–**F**) Corresponding SEM images showing particle growth at the beginning of the reaction and during its progression at intermediates S1, S4, and S5.

In sample S3, the particles adopt an oval morphology with clearly defined phase boundaries (Figure 3A,B). Although both domains appear similar in surface topography, one side is slightly more bulbous, suggesting that the smaller region grows outward from the larger domain. Raman imaging (Figure 3C) confirms the presence of two chemically distinct phases, resolved via True Component Analysis (WiTec Project 5.3) [35,36,37,38]: the larger domain consists mainly of PS, while the smaller protruding region corresponds to p(GMA-*co*-EDMA). From this stage onward, phase separation is spectrally distinct. As the polymer network forms, the p(GMA-*co*-EDMA) phase becomes insoluble in the PS matrix, resulting in two domains with defined boundaries. This process continues until phase inversion occurs, first observed in sample S4 after 45 min at 70 °C (Figure 3D,E). The formation of these Janus-like intermediates can therefore be understood as a consequence of kinetically controlled phase separation: polymerization initially occurs within the swollen PS matrix, followed by segregation of the increasingly crosslinked p(GMA-*co*-EDMA) phase. The balance between polymer growth, diffusion, and interfacial tension governs the evolution from biphasic particles to the final porous spherical morphology. At this point, the two domains exhibit distinct morphologies: the elongated, narrow region is porous, whereas the broader cap remains non-porous. In SEM images, porous p(GMA-*co*-EDMA) appears darker due to diffuse electron scattering from its irregular surface, while smooth PS appears brighter because of strong backscattering. Raman imaging corroborates the increasing volume of the p(GMA-*co*-EDMA) domain (Figure 3F). As polymerization continues, the p(GMA-*co*-EDMA) domain expands and the PS cap progressively diminishes. After 60 min (S5), the particles appear spherical and monodisperse (Figure 3G–I). The PS layer becomes thinner, cracked, and distributed non-uniformly across the surface, consistent with gradual dissolution of the PS domain. Figure 6A–C illustrate the particle growth by showing how individual PS seeds organize as a monolayer on the GMA/EDMA droplet. The rendered images visualize the progressive densification and growth of particles as polymerization proceeds. In particular, the rendered images of S4 and S5 (Figure 6B,C) reveal the formation of larger, partially merged particles, indicating pronounced particle coalescence at advanced reaction stages. Corresponding SEM images (Figure 6D–F) corroborate these findings, revealing a clear morphological transition from loosely associated seeds to compact, partially fused assemblies. These structural changes indicate that particle coalescence and crosslinking progressively drive the formation of a stable porous framework.

After 24 h (S6), the particles appear predominantly porous, with only a flattened PS cap remaining (Figure 3K). The cap exhibits a concave indentation toward the particle interior, indicating partial dissolution of PS during the reaction. In Raman images, the cap appears pink (blue + red), indicating a mixture of PS and p(GMA-*co*-EDMA), consistent with its thinness in this region (Figure 3L). Compared to S5, additional small, spherical, non-porous PS particles are present, which accumulate primarily on the PS cap and appear to be expelled during the reaction. In some cases, the PS cap is absent, leaving a cavity at this position. The small PS spheres adhering to the cap likely represent expelled material, consistent with the overall reduction in PS content relative to S5. The controlled isolation of these intermediate structures provides new opportunities for particle design. For example, intermediates such as S4 can be used as starting materials for a second swelling–polymerization step, enabling the production of double- or multi-compartment particles. By varying monomers, porogens, and reaction conditions, a broad range of Janus particles with different surface textures and physical properties can be synthesized [24]. Furthermore, the intermediates could serve as templates for silica particles, which have numerous potential applications [39,40,41,42,43,44,45,46]. Although the presented synthesis provides detailed insight into particle formation and enables access to anisotropic intermediates, its transfer toward industrial-scale production requires further optimization. The current seed swelling polymerization relies on precise control of parameters such as monomer diffusion, phase separation, and interfacial interactions, which may become more challenging upon scale-up. Potential strategies to improve the practical applicability include optimization of reaction conditions, improved control of mixing and heat transfer, and systematic adjustment of monomer, porogen, and stabilizer concentrations to ensure reproducible particle morphology and pore structure. These improvements could facilitate the future translation of this synthesis concept toward larger-scale production of functional porous polymer particles.

## 5. Conclusions

In this study, we investigated the formation mechanism of porous anisotropic p(GMA-*co*-EDMA) particles based on non-porous PS seeds. While current theory generally assumes that PS particles remain individually dispersed in solution until polymerization initiates, our results reveal a different scenario: after swelling with the monomer emulsion (S1), the PS seeds assemble into hollow spherical agglomerates composed of lenticular particles. Upon heating to 70 °C, polymerization begins, and from sample S3 onward, JPs emerge as intermediates with a porous p(GMA-*co*-EDMA) domain and a non-porous PS cap. The main polymerization and network formation is essentially complete after 1 h (S5), with only minor particle growth and further reduction in the PS fraction occurring over the subsequent 23 h until S6 (Supporting Information, Table S1).

These observations demonstrate that particle-particle interactions, interfacial localization, and kinetically controlled phase separation occur much earlier than generally considered in conventional seed swelling polymerization models, collectively governing the transition from swollen PS seeds to anisotropic intermediates and finally to homogeneous porous particles. Ultimately, the reaction yields homogeneous porous particles with a narrow size distribution and high porosity. The results highlight the role of emulsion droplets, monomer diffusion, and phase separation in directing particle morphology, providing mechanistic insight into the formation of porous and anisotropic structures. The results of this work now enable the rapid production of JPs under simple reaction conditions by quenching the reaction at defined time points. The identified Janus-like intermediates provide a versatile platform for the generation of anisotropic polymer particles with tailored properties. Due to the presence of functional groups within the p(GMA-*co*-EDMA) phase, these intermediates offer opportunities for subsequent modification and application-specific design. Furthermore, controlled interruption of the polymerization process enables access to defined intermediate structures, providing new possibilities for the synthesis of compartmentalized and multifunctional particles.

## Figures and Tables

**Table 1:** Values for GMA:EDMA (C1) and PS (C2) corresponding to the spectra shown in Figure 4A.

Sample	PS (C1) in (%)	GMA:EDMA (C2) in (%)
S1	99.1	0.9
S2	99.3	0.7
S3	94.4	5.6
S4	68.2	31.8
S5	48.8	51.2
S6	35.7	64.3

## Data Availability

The original contributions presented in this study are included in the article and supplementary material. Further inquiries can be directed to the corresponding authors.

## Supplementary Materials

The following supporting information can be downloaded at: https://www.mdpi.com/article/10.3390/polym18151813/s1, Table S1: Statistical parameters of the particle diameters, including the total number of analyzed particles as well as the mean, median, standard deviation, minimum, and maximum values. Figure S1: Confocal Raman images of S1 and S2 (A) and S3, S4, S5 and S6 (B). The yellow cross in the respective image shows the location on the particle that belongs to the Raman spectrum which is given below the image. The spectra given are the unprocessed raw spectra (not smoothed and not baseline corrected) just the cosmic events have been removed. PS parts are shown in red, whereas the blue particle part represents p(GMA-*co*-EDMA). The spectra of S1 and S2 do not differ from PS spectrum (Figure 1). At this point, no p(GMA-*co*-EDMA) can be detected spectrally in the particles. From sample S3 onwards poly (GMA-*co*-EDMA) (blue) can be found for the first time and clearly assigned in confocal Raman image. In S6, the spectrum of the overlapping area from PS and p(GMA-*co*-EDMA) is shown. Figure S2: SEM images of Intermediates (I1–I3) are shown, which occur between S1 und S2. The upper row of images (A,C,E,G,I) displays the overview SEM images of the intermediates, S1 and S2 whereas the lower row (B,D,F,H,J) shows an enlargement of the corresponding sample. Time and temperature at which the samples (S1, S2, I1–I3) were collected are given below the respective image section. Up to I3 (t = 34 min, T = 70 °C) all samples consist of spherical agglomerates of lens-shaped particles with various sizes and individual lenses that do not differ in size. Within one minute at the transition from I3 (t = 34 min, T = 70 °C) to S2 (t = 35 min, T = 70 °C) all particles change their shape and become spherical. Figure S3: Particle size distributions of the particle diameters (µm) for PS (red), S1 (green), and S6 (blue). The PS seed particles exhibit the smallest dispersion, with all particle diameters falling within the range of 2.0–2.3 µm. The size distribution of the lens-shaped particles in S1 shows a larger full width at half maximum (FWHM). The largest spread is observed for the final particles (S6), where distinctly larger particles are occasionally found within an otherwise monodisperse population. Particle diameters were manually measured from microscopy images using the image analysis software ImageJ. Statistical parameters including mean, median, standard deviation, as well as minimum and maximum particle diameters were determined using the same software. All relevant statistical parameters are summarized in Table S1.

## Abbreviations

The following abbreviations are used in this article:

p(GMA-*co*-EDMA)	poly(glycidyl methacrylate-co-ethylene dimethacrylate)
SEM	scanning electron microscopy
GMA	glycidyl methacrylate
EDMA	ethylene dimethacrylate
PS	polystyrene
RED	relative energy difference
JP	Janus particle
