# Supplementary material for: Mechanistic Insights into the Formation of Monodisperse Porous Poly(GMA-co-EDMA) Microspheres
Source: Polymers (Basel). 2026 Jul 24;18(15):1813. doi: 10.3390/polym18151813 (PMC13468068; doi:10.3390/polym18151813)
Supplement: Supplementary file 1 [file polymers-18-01813-s001.zip › polymers-4441639-supplementary.pdf]

Supplementary

Materials and Methods

Preparation of monodisperse polystyrene (PS) seeds

Polystyrene (PS) microspheres (2.13 ± 0.06 µm) were synthesized in our laboratory using dispersion polymerization in alcoholic media. 20 mL styrene (99 %) and 80 mL ethanol (96 %) were added in a three-necked round-bottom flask (250 mL). After the addition of 1 g PVP as stabilizer and 0.5 g BPO as initiator, the suspension was sonicated for 10 min. The suspension was stirred at 120 rpm with a magnetic stirrer at room temperature for 30 min while purging with Argon. Thereafter, the temperature was increased to 70 °C for 24 h under reflux. The PS seed particles were centrifugated for 2 min at 7500 rpm and were washed three times with ethanol and three times with deionized water to remove the reaction solution. The particles were freeze-dried under vacuum for 72 h.

How were the colours assigned to the particles?

A principal component analysis (PCA) of all Raman spectra yielded two principal components. Based on this number of relevant components, the data were analysed with Control 5.3 data processing software provided by WITec using an inbuilt PCA. The components were assigned to colours (blue, red) for better visualization. Regions dominated by polystyrene spectra were assigned the colour red, while those dominated by EDMA/GMA were assigned the colour blue.

Particle characterisation

Table S1: Statistical parameters of the particle diameters, including the total number of analyzed particles as well as the mean, median, standard deviation, minimum, and maximum values.

Sample	Time	N total	Mean Diameter (µm)	Median Diameter (µm)	Standard deviation	Minimum Diameter (µm)	Maximum Diameter (µm)
**PS**	-	502	2.13	2.13	0.06	2.0	2.30
**S1**	Start	501	3.02	3.02	0.12	2.50	3.40
**S2**	35 min	800	2.76	2.43	1.31	2.15	16.05
**S3**	40 min	1000	2.84	2.60	0.52	2.52	4.48
**S4**	45 min	500	3.48	3.48	0.10	3.30	3.66
**S5**	60 min	500	5.88	5.87	0.19	5.56	6.20
**S6**	24 h	500	6.38	6.39	0.19	5.13	6.39

Raman spectra S1 - S6

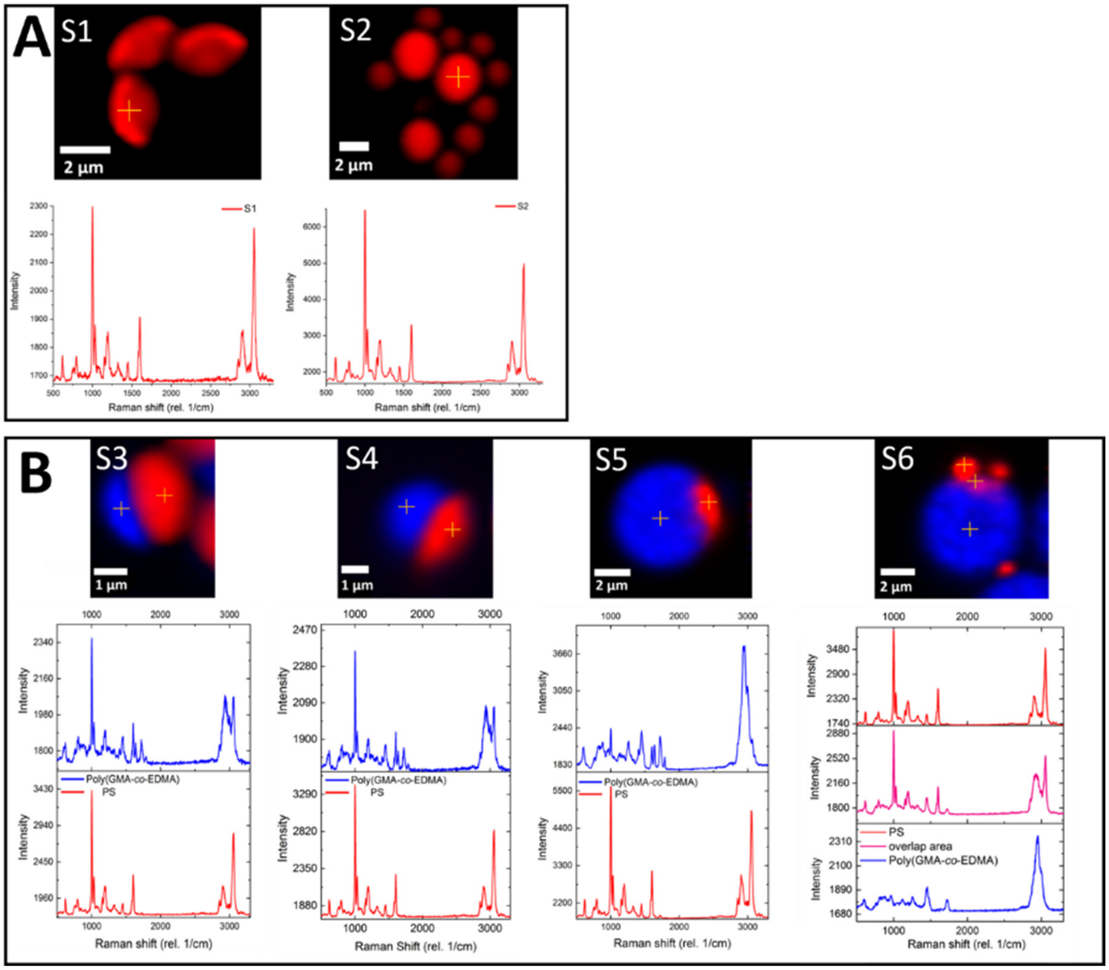

Figure S1: Confocal Raman images of **S1** and **S2** (A) and **S3**, **S4**, **S5** and **S6** (B). The yellow cross in the respective image shows the location on the particle that belongs to the Raman spectrum which is given below the image. The spectra given are the unprocessed raw spectra (not smoothed and not baseline corrected) just the cosmic events have been removed. PS parts are shown in red, whereas the blue particle part represents p(GMA-*co*-EDMA). The spectra of **S1** and **S2** do not differ from PS spectrum (Figure 1). At this point, no p(GMA-*co*-EDMA) can be detected spectrally in the particles. From sample **S3** onwards poly (GMA-*co*-EDMA) (blue) can be found for the first time and clearly assigned in confocal Raman image. In **S6**, the spectrum of the overlapping area from PS and p(GMA-*co*-EDMA) is shown.

SEM images of intermediate steps between S1 and S2

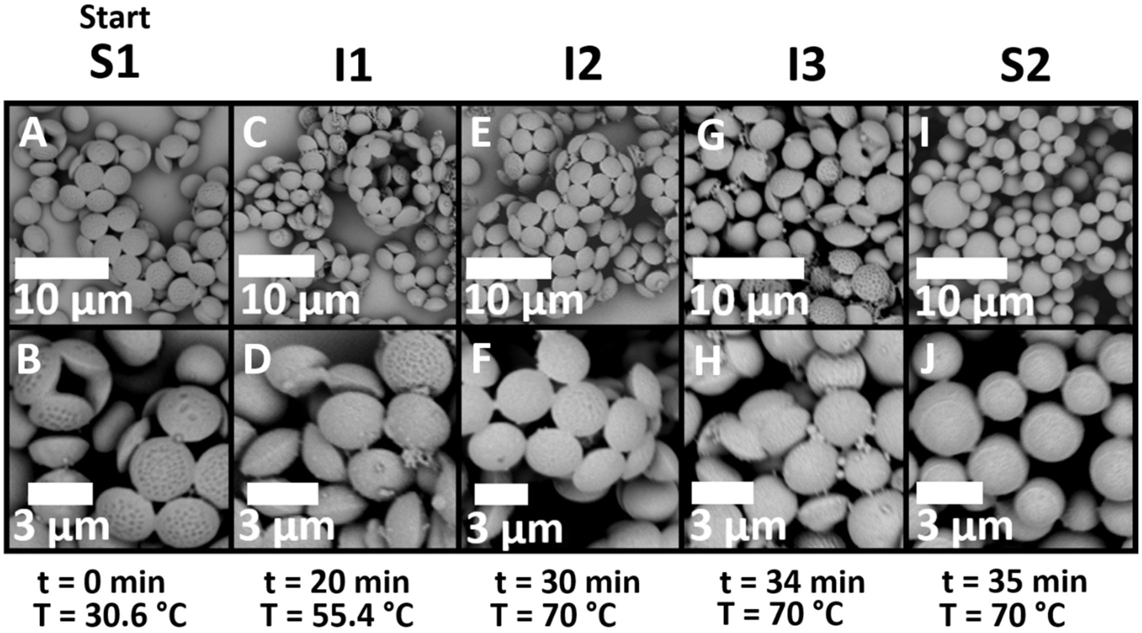

Figure S2: SEM images of Intermediates (**I1** - **I3**) are shown, which occur between **S1** und **S2**. The upper row of images (A, C, E, G, I) displays the overview SEM images of the intermediates, **S1** and **S2** whereas the lower row (B, D, F, H, J) shows an enlargement of the corresponding sample. Time and temperature at which the samples (**S1**, **S2**, **I1** - **I3**) were collected are given below the respective image section. Up to **I3** (t = 34 min, T = 70 °C) all samples consist of spherical agglomerates of lens-shaped particles with various sizes and individual lenses that do not differ in size. Within one minute at the transition from **I3** (t = 34 min, T = 70 °C) to **S2** (t = 35 min, T = 70 °C) all particles change their shape and become spherical.

Particle size distribution

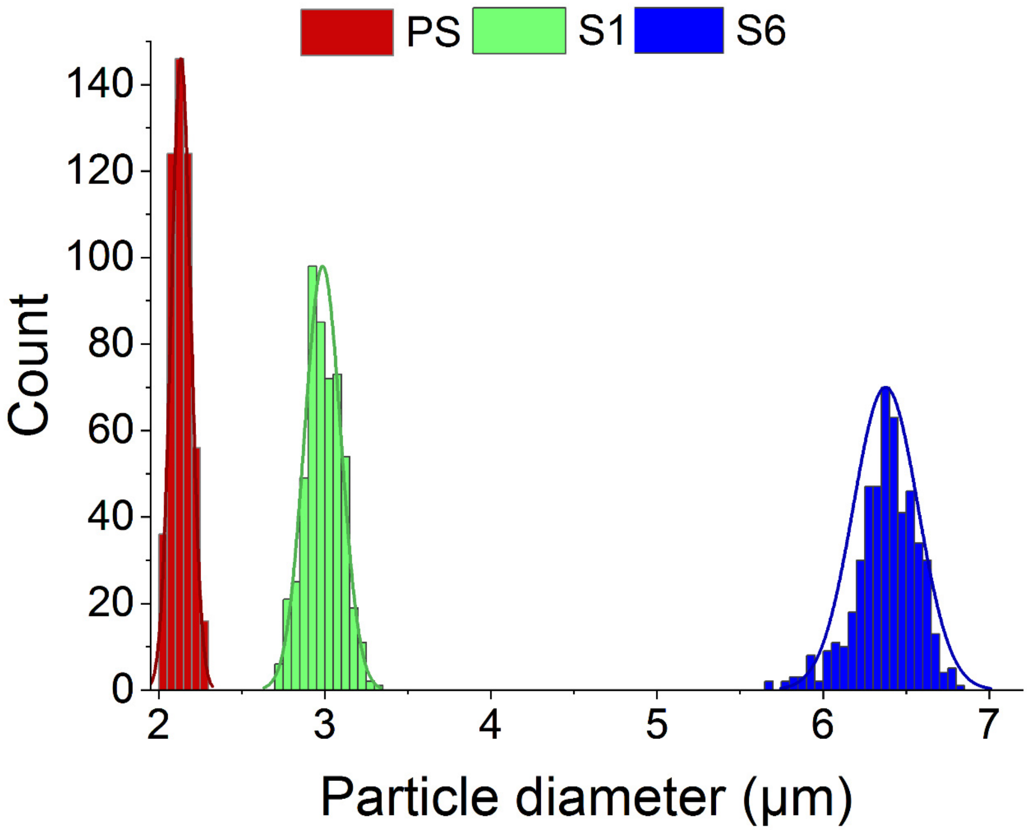

Figure S3: Particle size distributions of the particle diameters (µm) for PS (red), S1 (green), and S6 (blue). The PS seed particles exhibit the smallest dispersion, with all particle diameters falling within the range of 2.0–2.3 µm. The size distribution of the lens-shaped particles in S1 shows a larger full width at half maximum (FWHM). The largest spread is observed for the final particles (S6), where distinctly larger particles are occasionally found within an otherwise monodisperse population. Particle diameters were manually measured from microscopy images using the image analysis software **ImageJ**. Statistical parameters including mean, median, standard deviation, as well as minimum and maximum particle diameters were determined using the same software. All relevant statistical parameters are summarized in Table S1.
